# Noncanonical Inflammasomes: Caspase-11 Activation and Effector Mechanisms

**DOI:** 10.1371/journal.ppat.1003144

**Published:** 2013-02-28

**Authors:** Petr Broz, Denise M. Monack

**Affiliations:** 1 Focal Area Infection Biology, Biozentrum, University of Basel, Basel, Switzerland; 2 Department of Microbiology and Immunology, Stanford School of Medicine, Stanford University, Stanford, California, United States of America; University of North Carolina at Chapel Hill School of Medicine, United States of America

## Introduction

Inflammasomes are cytosolic, multiprotein complexes assembled by members of the NOD-like receptor (NLR) and PYHIN protein families in response to pathogen-associated molecular patterns (PAMPs) and danger signals, and serve as activation platforms for caspase-1. Recently, a new noncanonical inflammasome pathway has been described that activates caspase-11, an understudied pro-inflammatory caspase. Despite new insights into the signaling events that control caspase-11 activation, a number of unanswered questions remain.

## What Are the Signals That Trigger Noncanonical Inflammasome Activation?

Activation of the noncanonical inflammasome pathway has been observed in response to a number of Gram-negative bacteria (*Citrobacter rodentium*, *Escherichia coli*, *Vibrio cholerae*, *Salmonella typhimurium*, and others), but not with Gram-positive bacteria [1]–[3]. This distinction indicates that maybe lipopolysaccharide (LPS), a component of the outer membrane of Gram-negative bacteria, could be the activator of caspase-11. Nevertheless, although LPS plays an important part in the activation of the noncanonical inflammasome [2], [3], LPS alone is not sufficient to activate this pathway (discussed in detail below), indicating that an additional bacteria-derived signal must exist.

In this regard, it is intriguing that cholera toxin B (CTB) together with LPS was shown to be another activator of caspase-11 [1]. Although membrane damage could be a signal for caspase-11 activation, it is unlikely since other pore-forming toxins such as *Clostridium difficile* toxin B, listeriolysin O, and adenylcyclase (AC) toxin do not activate the noncanonical pathway [1]. Another possible activator would be bacterial RNA, which was recently proposed to trigger IL-1β maturation and cell death during *E. coli* or *S. typhimurium* infections. Interestingly, inflammasome activation by bacterial RNA required Trif (TIR-domain-containing adaptor inducing interferon-β) and NLRP3 [4], which are also involved in noncanonical inflammasome activation [1]–[3]. However, the observation that LPS alone is sufficient to induce caspase-11-dependent septic shock in vivo [1] would argue against a role for bacterial RNA. Thus, further experiments will be required to identify the bacterial signals that trigger the noncanonical inflammasome pathways and to understand the roles of LPS and bacterial RNA in this process.

## How Does Canonical and Noncanonical Inflammasome Signaling Differ?

**Figure 1:**
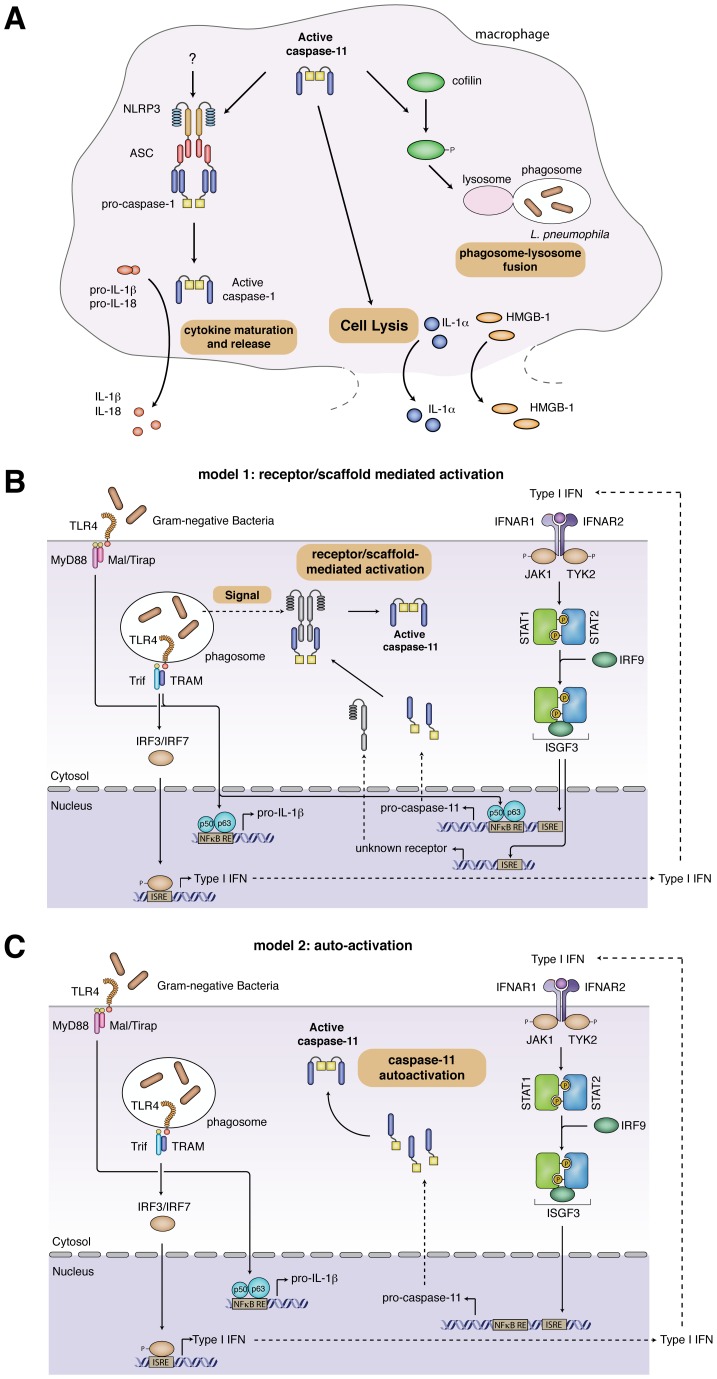
Caspase-11 effector functions and models for caspase-11 activation. (**A**) **Caspase-11 effector functions.** Active caspase-11 cooperates with components of the NLRP3 inflammasome to induce caspase-1-dependent maturation of pro-IL-1β and pro-IL-18. It remains to be determined if caspase-11 activates NLRP3 directly or if additional signals are required. Active caspase-11 also induces cell lysis, resulting in the release of danger signals such as IL-1α and HMGB-1. Finally, during *L. pneumophila* infections, caspase-11 controls phagosome-lysosome fusion through the phosphorylation state of cofilin. (**B, C**) **Two distinct models for caspase-11 activation.** (B) *Receptor/scaffold-mediated activation*. Detection of Gram-negative bacteria by TLR4 results in the activation of NFκB and subsequent expression of pro-IL-1β and pro-caspase-11. Signaling through Trif and IRF3 induces expression of type-I-IFNs. Type-I-IFN signaling through IFNαR contributes to pro-caspase-11 expression and induces the expression of an uncharacterized receptor/activator of caspase-11. Activation of caspase-11 by this factor might require an additional undefined signal, stemming from the bacterial infection. (C) *Autoactivation of pro-caspase-11*. Detection of Gram-negative bacteria by TLR4 results in the activation of NFκB and subsequent expression of pro-IL-1β. Signaling through Trif and IRF3 induces expression of type-I-IFNs, which induces pro-caspase-11 expression. Pro-caspase-11 autoactivates, presumably once a concentration threshold is reached.

Although both caspase-1 and caspase-11 eventually initiate cell lysis and the release of processed cytokines and danger signals, the hallmarks of inflammasome signaling [5], their underlying mechanisms differ significantly (Figure 1A). Caspase-1 activation by canonical stimuli induces a pro-inflammatory, lytic cell death called pyroptosis. Although caspase-11 activation also induces lysis of the host cell, caspase-11-dependent cell death has features that distinguish it from pyroptosis. Pyroptosis is accompanied by the release of mature, processed cytokines (IL-1β and IL-18) that are secreted by a caspase-1-dependent mechanism called unconventional secretion [6]. In contrast to this, caspase-11 lacks the ability to cleave these cytokines by itself, since macrophages deficient in *Nlrp3*, *ASC*, or *Casp1* still activate caspase-11 and initiate cell death but do not release mature IL-1β or IL-18. This suggests that caspase-11 acts in conjunction with the NLRP3 inflammasome to promote cytokine maturation [1]. The exact mechanism of this interaction is controversial, which in part could be accounted for by the different assays that have been used to monitor NLRP3 inflammasome assembly. Microscopic analysis of ASC speck formation suggests that caspase-11 acts upstream of NLRP3 [2], which is consistent with observations reported by the Yuan group [7], while biochemical enrichment of inflammasomes indicates that caspase-11 is downstream of NLRP3 [3]. In conclusion, since caspase-11-mediated cell death lacks associated cytokine maturation, it resembles a programmed lytic cell death more like necroptosis than pyroptosis.

Another difference between canonical and noncanonical inflammasomes is in the release of IL-1α and the danger signal high-mobility group box 1 (HMGB1). The release of IL-1α and HMGB1 by canonical inflammasomes requires active caspase-1-mediated secretion [6], while caspase-1 is not required for their release in response to CTB and *E. coli* [1], suggesting that lysis is the release mechanism for these factors following caspase-11 activation (Figure 1A). Whether caspase-11 also initiates novel caspase-1 effector mechanisms like the release of eicosanoids [8] and the secretion of growth factors [5] remains to be determined. Further work will be required to identify and characterize additional effector mechanisms of caspase-11 in vitro, such as phagosome-lysosome fusion (discussed separately below) [9], and to determine how these affect the role of caspase-11 in pathogenesis in vivo.

## How Is Pro-Caspase-11 Expression Controlled?

Resting macrophages or dendritic cells (DCs) express very low levels of pro-caspase-11. The pro-caspase-11 promoter contains NFκB and STAT binding sites, and expression is highly inducible by LPS, IFN-γ and TNFα treatment [10]. Recently, we and others have linked caspase-11 expression and activation to the signaling through Toll-like receptor 4 (TLR4) and Trif [2], [3]. The induction of pro-caspase-11 mRNA and protein expression is significantly delayed in macrophages from *TLR4^−/−^* or *Trif^−/−^* mice following infection with *S. typhimurium* ([2] and unpublished results). Signaling via MyD88 is also involved, since *MyD88*-deficient macrophages show a slight delay in pro-caspase-11 induction. Nevertheless, pro-caspase-11 expression was not totally abolished in *MyD88^−/−^/Trif^−/−^* macrophages, suggesting that additional pathways contribute to the transcriptional induction of *Casp-11* [2]. Similarly, Rathinam et al. show that LPS treatment or EHEC infections result in lower levels of pro-caspase-11 induction in *Trif^−/−^* macrophages [3]. Unexpectedly, their results do not show a contribution of MyD88 to caspase-11 induction, but their study did not directly compare *Trif^−/−^* to *MyD88^−/−^/Trif^−/−^* macrophages [3].

Trif-dependent induction of pro-caspase-11 expression could occur either by activating NFκB or through IRF3-mediated production of type-I-interferon (type-I-IFN). *Irf3*, *Ifnar1 (interferon-α/β receptor)*, or *STAT-1* deficiency delays pro-caspase-11 induction in *S. typhimurium*–infected macrophages, suggesting that the type-I-IFN pathway contributes to pro-caspase-11 expression. However, pro-caspase-11 expression is not completely abolished in the absence of type-I-IFN signaling [2]. In contrast, Rathinam et al. report that *Ifnar1* deficiency completely abolishes pro-caspase-11 expression in response to LPS, IFN-β, or EHEC infection [3]. In addition, they show that IFN-β treatment significantly increases pro-caspase-11 expression compared to mock-treated macrophages [3], which is consistent with the observation that IFN-β treatment slightly increases pro-caspase-11 expression in DCs [11]. Conversely, IFN-β treatment of macrophages does not enhance pro-caspase-11 levels during *Salmonella* infections [2]. In conclusion, different pathogens or stimuli seem to induce pro-caspase-11 expression via distinct signaling pathways. Since the pathways that lead to induction of pro-caspase-11 expression are likely important for the different models of caspase-11 activation (discussed below), further analysis of caspase-11 induction is required.

## What Is the Mechanism of Caspase-11 Activation?

TLR4/Trif-mediated type-I-IFN production is essential for caspase-11 activity; macrophages deficient in *Tlr4*, *Trif*, *Irf3*, *ifnar*, *STAT-1*, or *Irf9* do not initiate cell death and the release of processed caspase-11 and cytokines in response to noncanonical inflammasome stimuli [2], [3]. However, the mechanism through which IFN-β controls caspase-11 activation remains controversial, and two opposing models have been proposed (Figure 1B, C).

One model for caspase-11 activation suggests a receptor/scaffold-mediated activation mechanism. We observed that signaling via IFNαR and STAT-1 is crucial for caspase-11 activity in macrophages infected with *S. typhimurium* [2], yet this does not result from a lack of pro-caspase-11 expression, since significant levels of pro-caspase-11 are present in cells deficient for components of the type-I-IFN signaling cascade. Consistently, *MyD88^−/−^/Trif^−/−^* macrophages, which are deficient for type-I-IFN production, do express pro-caspase-11 when infected with *S. typhimurium*, but do not activate caspase-11. Restoring type-I-IFN signaling by adding exogenous IFN-β rescues this defect in *Salmonella*-infected *MyD88^−/−^/Trif^−/−^* macrophages, and this is independent of pro-caspase-11 induction [2]. Importantly, treatment with exogenous type-I-IFN in the absence of an infection does not result in caspase-11 activation in primary BMDMs, as exemplified by a lack of active caspase-11 p30 in the cell supernatant and absence of cell death [2]. These results indicate that in primary macrophages type-I-IFN is required for the expression of an interferon-inducible activator or receptor for caspase-11 (Figure 1B). They also raise the possibility that a yet-unknown bacterial signal is required for the activation of caspase-11.

In contrast to the receptor/scaffold-mediated activation mechanism, Rathinam et al. have suggested that pro-caspase-11 expression is both necessary and sufficient to induce pro-caspase-11 autoactivation (Figure 1C) [3]. This conclusion was based on the observation that treating macrophages with LPS, IFN-β, and IFN-γ for 16 h simultaneously induced pro-caspase-11 expression and activated caspase-11, as judged by the appearance of a processed caspase-11 p30 band in the lysates of these cells. However, the significance of this processed cytoplasmic caspase-11 is unclear, since the authors did not provide corresponding cell-death data for this timepoint and did not show the release of the caspase-11 p30 subunit into the cell supernatant [3]. To support the model, Rathinam et al. showed that IFN-β and IFN-γ treatment indeed induced cell death, but only at far later timepoints (40 h posttreatment) and in BMDMs immortalized with v-myc/v-raf–expressing J2 retrovirus [3]. However, they did not show if LPS treatment also induces cell death at 40 h in immortalized cells. It must be noted though that data obtained with immortalized macrophages have to be interpreted carefully, since these cells are known to produce replication-proficient viruses (unpublished observation).

In conclusion, since LPS pretreatment is routinely used to prestimulate macrophages and was reported not to induce cell death even as late as 24 h poststimulation [4], it is unlikely that LPS treatment alone is sufficient to activate caspase-11. Similarly, IFN-β treatment in the absence of infection does not induce cell death in primary cells [12].

Another argument brought forward in support of autoactivation is the observation that expression of pro-caspase-11 in 293T cells or cell-free systems results in autoprocessing of caspase-11. However, the usefulness of these systems for the study of caspase activation is limited, since even caspase-1 (which is activated in a receptor-mediated manner) autoactivates in the 293T cell expression system [13], [14]. In addition, pro-caspase-11 expression has been successfully restored in *casp1/casp11*-deficient macrophages, and autoactivation has not been reported [9], [15].

Given the importance of caspase-11 in pathogenesis, a better understanding of the mechanism of caspase-11 activation is definitely required. The identification of a specific caspase-11 receptor and/or a bacterial ligand (other than LPS) required for caspase-11 activity would resolve this issue.

## What Is the Role of Caspase-11 in Phagosome Maturation?

The Nlrc4/Naip5 inflammasome restricts the replication of intracellular *Legionella pneumophila* by activating caspase-1 and caspase-7. Caspase-11 was recently shown to also restrict the growth of *Legionella* in macrophages and in the lungs of infected mice [9]. Since this was also dependent on a functional dot/Icm system, NLRC4, and flagellin, the authors of that study have suggested that NLRC4 could activate caspase-11 [9], which is in contrast to data reported previously [1]. Finally, the authors showed that caspase-11 might promote the fusion of the *L. pneumophila* vacuole with lysosomes by modulating actin polymerization through cofilin (Figure 1A). These results suggest that caspase-11 has other effector mechanisms besides cell death and NLRP3/caspase-1-dependent cytokine maturation. However, the modulation of phagosome-lysosome fusion might be specific for *L. pneumophila* infections, since caspase-1, -7, or -11-dependent growth restriction has so far not been reported for other bacteria activating the noncanonical inflammasome [2], [3].

## What Are the Functions of Caspase-11 In Vivo?

The noncanonical inflammasome pathway has been shown to be activated by a range of different Gram-negative bacteria but not by Gram-positive bacteria, suggesting that it is a conserved mechanism for the detection of these pathogens. But it is less evident how the activation of caspase-11 benefits the host in the context of an infection. Growth restriction has been shown to control bacterial number in the lungs of mice infected with *L. pneumophila* [9]; however, caspase-11-mediated cell death often results in detrimental effects for the host. For example, caspase-11-mediated cell lysis was responsible for the lethal effects of LPS in a mouse model of sepsis, which occurred independently of NLRP3, ASC, and caspase-1, thus excluding an involvement of cytokine production [1], [7]. In addition, caspase-11-mediated cell death increased susceptibility of mice to *S. typhimurium* infections in the absence of caspase-1 [2]. This result was surprising, since caspase-1-induced pyroptosis is important for the clearance of intracellular bacterial pathogens [16]. Further analysis showed that in the absence of caspase-1-initiated innate immune defenses, caspase-11-dependent cell lysis promotes spread and extracellular replication of *Salmonella*, a facultative intracellular bacterial pathogen. It is conceivable that caspase-11 evolved to support caspase-1 by providing additional means of inducing cell lysis. But since caspase-11 has lost the ability to promote cytokine maturation, caspase-11 activation can be exploited by intracellular pathogens as a silent way to egress from infected host cells and to spread within the host. Future work will address how caspase-11 supports innate immune defenses against other Gram-negative bacterial pathogens or whether caspase-11 has detrimental effects for the host in other infectious disease models.
